# G-Protein Phosphorylation: Aspects of Binding Specificity and Function in the Plant Kingdom

**DOI:** 10.3390/ijms23126544

**Published:** 2022-06-11

**Authors:** Celio Cabral Oliveira, Alan M. Jones, Elizabeth Pacheco Batista Fontes, Pedro A. Braga dos Reis

**Affiliations:** 1 Department of Biochemistry and Molecular Biology, Universidade Federal de Viçosa, Viçosa 36570, Brazil; celio.oliveira@ufv.br (C.C.O.); bbfontes@ufv.br (E.P.B.F.); 2 National Institute of Science and Technology in Plant-Pest Interactions, Bioagro, Viçosa 36570, Brazil; 3 Department of Biology, University of North Carolina at Chapel Hill, Chapel Hill, NC 27599, USA; alanjones@bio.unc.edu; 4 Department of Pharmacology, University of North Carolina at Chapel Hill, Chapel Hill, NC 27599, USA

**Keywords:** phosphorylation, G protein, RGS, GPA1, AGB1, XLG, AGG, kinase, structure, regulation

## Abstract

Plant survival depends on adaptive mechanisms that constantly rely on signal recognition and transduction. The predominant class of signal discriminators is receptor kinases, with a vast member composition in plants. The transduction of signals occurs in part by a simple repertoire of heterotrimeric G proteins, with a core composed of α-, β-, and γ-subunits, together with a 7-transmembrane Regulator G Signaling (RGS) protein. With a small repertoire of G proteins in plants, phosphorylation by receptor kinases is critical in regulating the active state of the G-protein complex. This review describes the in vivo detected phosphosites in plant G proteins and conservation scores, and their in vitro corresponding kinases. Furthermore, recently described outcomes, including novel arrestin-like internalization of RGS and a non-canonical phosphorylation switching mechanism that drives G-protein plasticity, are discussed.

## 1. Introduction

**Figure 1:**
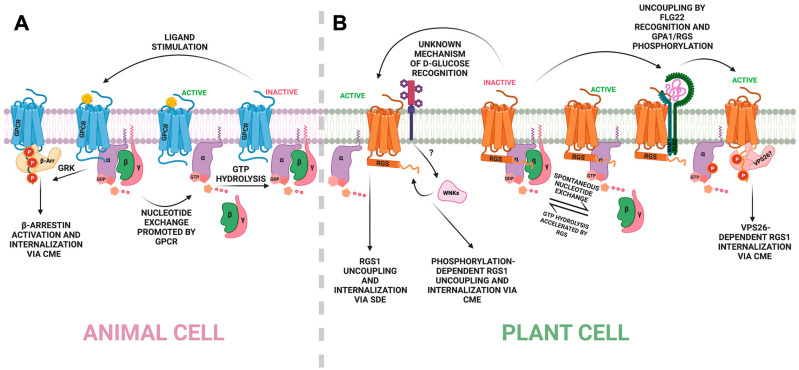
Conserved and non-conserved G-protein activation mechanisms in plants and animals. (**A**) An animal cell recognizes an extracellular signal via GPCR that promotes nucleotide exchange at the alpha subunit. GTP-bound Gα releases Gβγ for downstream signaling. Inactivation occurs under GTP hydrolysis and phosphorylation-induced GPCR internalization. (**B**) Nucleotide exchange is spontaneous in plant cells with no characterized GPCR. Negative regulation via GTPase acceleration activity is promoted by 7TM-RGS proteins. D-glucose activates endocytosis via two different mechanisms: RGS1 is phosphorylated by the WNKs and internalized in a VPS26-independent module via clathrin-mediated endocytosis (CME), or RGS1 is internalized in a phosphorylation-independent mechanism via sterol-dependent endocytosis (SDE). Flg22 is recognized by the BAK1/FLS2 complex, and multiple phosphorylation occurs at GPA1 and at the C-terminus of RGS1. The phosphorylated core is uncoupled, and downstream signaling is activated. Flg22-induced RGS1 internalization occurs via CME in a β-arrestin-like mechanism mediated by the VPS26 proteins. Created with BioRender.com (Publication license OL240ET01G. Accessed on 7 June 2022).

Plants lack the mobility mechanisms observed in other kingdoms; hence, their survival depends on adaptive mechanisms that constantly rely on signal perception and transduction [1]. Among the main signaling molecules, the heterotrimeric G proteins play an essential role. They are composed of α-, β-, and γ-subunits, modulated by nucleotide-binding status. The activation/inactivation of the complex occurs through the GTP/GDP binding process. The Gα-GDP binding maintains the complex in an inactive form, and Gα remains associated with Gβ and Gγ proteins. During the activation process, GDP is replaced by GTP, which promotes the dissociation of Gα from Gβγ proteins and, in turn, triggers the downstream signaling [2,3]. The modulation of the Gα protein to GDP-bound or GTP-bound is a precise and specific process. In mammals, the modulation of the GDP-to-GTP exchange mechanism is performed by G-protein-coupled receptors (GPCRs) that act as guanine nucleotide exchange factors (GEFs). Gα protein has intrinsic GTPase activity, but with a slow rate of hydrolysis. Therefore, it requires some factor(s) to accelerate the GTPase activity to modulate the signaling to a steady state (Figure 1) [4,5].

In metazoans, many molecules activate different pathways through G proteins [6]. The signal distinction is mainly explained by a vast combination of subunits and GPCRs present in their genome [7]. On the other hand, plant genomes encode a few subunits; for example, the *Arabidopsis thaliana* genome encodes only one canonical Gα subunit (AtGPA1), three atypical Gα subunits (AtXLG1-3), one beta (AtAGB1), and three gamma (AtAGG1-3) subunits, one RGS regulator (AtRGS1), and no characterized GPCR [8]. This limited number of protein components does not correlate with the complexity of the signaling events mediated by G protein in plants [9]. The multiplicity of propagated signals from plant G proteins relies on the different activator receptors and various post-translational modifications on the G subunits, rather than the number of components [10]. Moreover, in plants, algae, and protists, Gα presents GPCR-independent nucleotide exchange, and some species are heavily regulated by the atypical seven-transmembrane (7TM) Regulator of G-signaling 1 (RGS1) (Figure 1) [11,12,13]. As cytoplasmic kinases and receptor-like kinases (RLKs) are consistently linked to G-protein mediation [14,15,16], here, we discuss the mapping of phosphorylation events and outcomes regarding the G-signaling core in plants.

## 2. Phosphorylation and Internalization of RGS1 in an Arrestin-like Mechanism

G-protein-coupled receptors are composed of an extracellular N-terminus, a 7TM domain with intra- and extracellular loops, and a disordered cytoplasmic C-terminal domain [17]. GPCRs bind agonists, leading to activation through a conformational change that relays the signal to the Gα subunit regulation by accelerating the release of bound GDP [18]. GPCRs are phosphorylated by GPCR kinases (GRKs), initiating the recruitment and activation of adaptor proteins, β-arrestins, that precede clathrin binding and endocytosis [19]. Β-arrestins affect signaling by internalizing the GPCR away from its G-protein complex, and they also propagate signaling by interacting with effector proteins [20]. Different phosphorylation patterns at the V2 vasopressin receptor (V_2_R) C-tail promote different levels of β-arrestin1 binding and activation via conformational changes. Those findings suggest a “phosphorylation barcode” reading in which the spatial arrangement of phosphate groups determines the recruitment and activation of β-arrestins, rather than the number of phosphorylated residues at the receptor (Figure 1) [21].

**Figure 2:**
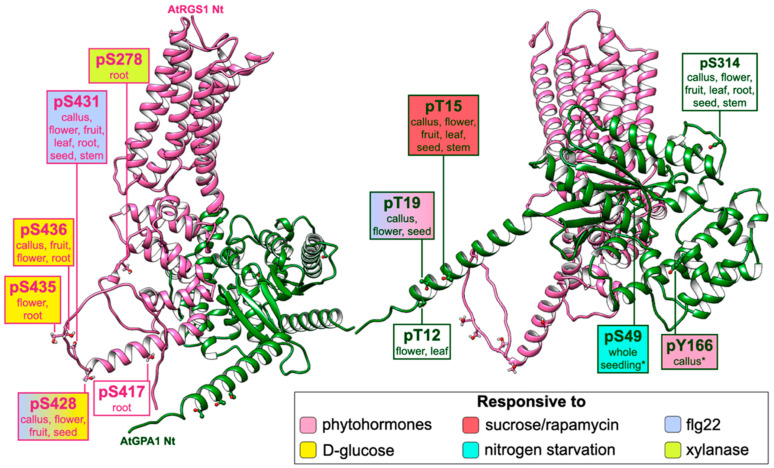
Experimental phosphorylation map of AtRGS1 and AtGPA1 dimer. Structural models of AtRGS1 (hot pink) and AtGPA1 (forest green) are shown. Xylanase-induced phosphorylation is detected at serine 278, which constitutes the linker region of AtRGS1 [25,26]. Phosphorylation occurs at the C-terminal tail of AtRGS1 in the serine residues 417, 428, 430, 431, 435, and 436 [26]. d-glucose-induced phosphorylation of AtRGS1 occurs at Ser428/435/436 [14], and phosphorylation under flg22 treatment is Ser428/431-dependent [27]. AtGPA1 is phosphorylated at the N-terminal threonine residues 12, 15, and 19 [26,28,29,30,31,32]. pThr19 has a reduced phosphorylation signal with flg22 treatment but is induced by ABA. Tyrosine residue 166 is at the all-alpha helical domain interface and responds to several phytohormones [29,33]. Phosphorylation occurs at the catalytic domain of the serine residues 49 and 314, and pSer49 is induced by sugar exposure [26,29,34]. Top-ranked models were obtained using AlphaFold2 [35], and the dimer complex was predicted by overlapping the models with the crystal structure of the heterodimeric complex of human RGS1 and activated Gi alpha 1 (PDB 2GTP). Phosphosites are represented as balls and sticks. Experimental data were obtained from both the PhosphAt database (https://phosphat.uni-hohenheim.de, accessed on 20 May 2022) and ATHENA (http://athena.proteomics.wzw.tum.de, accessed on 20 May 2022). ATHENA was used to identify tissue-specific phosphorylation, which is pointed out below residue identification. Color filling indicates experimental treatment. Asterisks indicate residues that were not mapped in all tissues.

The structure of the Arabidopsis regulator, AtRGS1, has a hybrid architecture of GPCR topology and an animal RGS protein [22]. The prototype contains a GPCR-like seven-transmembrane barrel domain at the N-terminus, followed by a disordered linker region that may contain a short helix, a conserved RGS domain, and an unstructured C-terminal tail, which harbors several di-serines typical of GPCRs (Figure 2) [23]. AtRGS1 undergoes endocytosis under high concentrations of D-glucose within a few minutes in a Gβ-dependent manner. The C-terminus of the 7TM regulator possesses a cluster of serine residues (Ser428, Ser430, Ser431, Ser435, and Ser436) that resemble the ones found in mammalian organisms. Although GRKs have not been identified in plant genomes, several WNKs (WITH NO LYSINE KINASE) [24] interact with AtRGS1 and phosphorylate the C-tail residues Ser428 and Ser435 or Ser436 in vitro. The inactivation of those phosphosites (Ser → Ala mutation) and the deletion of some *WNK* genes reduce glucose-induced internalization of AtRGS1 [14].

The peptide flg22, a bacterial elicitor of host responses, binds to its receptor FLS2 (FLAGELLIN-SENSITIVE 2) and co-receptor BAK1 (BRI1-ASSOCIATED RECEPTOR KINASE 1), leading to the induction of specific response genes, ROS production, and calcium signaling [36,37]. However, the deletion of the *AtRGS1* gene impairs the flg22-mediated responses, indicating a genetic interaction between *AtRGS1* and FLS2 signaling [27,38,39]. Furthermore, other biotic pathways (e.g., anti-fungal responses elicited by chitin) are affected by AtRGS1, and bacterial infection in *rgs1-2* plants is attenuated compared to that in the wild type [27]. Since flg22 and chitin act as external signals, it is reasonable to assume that elicitor-modulated RLKs interact with and phosphorylate AtRGS1. Accordingly, BAK1 and its interacting partners FLS2, BIK1, PEPR1, and BIR1 have been shown to phosphorylate RGS1 in vitro [40]. Furthermore, genetic and biochemical assays indicate that RLK BRI1-LIKE 3 (BRL3) also interacts with AtRGS1 to control ROS production and plant development during flg22 and sugar responses [38]. Phosphorylation of 7TM-RGS also occurs in soybean, where the Nod factor receptor 1 (NFR1) phosphorylates GmRGS2 in vitro to control nodule formation. Interestingly, three of the five NFR1-induced phosphorylated residues are localized at the predicted linker region of GmRGS2, and one of them (Ser277) is conserved in AtRGS1 (Ser278) (Table 1) [41]. Likewise, this linker residue has been shown to be phosphorylated in xylanase-treated root cell cultures [25].

While the C-terminal serine cluster phosphorylation in response to sugar and pathogens has been confirmed, the specific phosphorylation sites are still unclear because distinguishing the mass spectrometry (MS) signals of neighbor phosphoserines is not an easy task [14,27]. The inactivation of Ser431 alone (AtRGS1^S431A^) inhibits the C-terminal phosphorylation induced by flg22, Elf18, chitin, and Pep9. Flg22-induced dissociation of RGS1/XLG2 and RGS1/FLS2 complexes is also inhibited by a single Ser431 mutation, while a quadruple phosphomimetic mutation at the cluster (AtRGS1^S428/431/435/436D^) causes defective binding of both complexes [27]. 

Consistent with the GPCR internalization mechanism and the biased signaling theory, in which different signal/receptor interactions trigger different pathways [54], AtRGS1 is internalized by two phosphorylation-dependent endocytosis pathways. Flg22 induces AtRGS1 internalization via clathrin-mediated endocytosis (CME), while D-glucose triggers both CME and sterol-dependent endocytosis (SDE). The recruitment of the CME endocytic machinery towards GPCRs requires prior β-arrestin binding and activation, but plant genomes do not encode these proteins [55,56]. Nevertheless, Arabidopsis has three proteins with arrestin folds that bind as heterodimers to AtRGS1 and are required for endocytosis [55]. These include the vacuolar sorting proteins 26 (VPS26)—AtVPS26a, AtVPS26b, and AtVPS26-like components of the retromer [57], well-known in animals for their role in endosomal to plasma membrane anterograde trafficking [58]. VPS26 appears to moonlight as β-arrestins in plants, and because some GPCR endocytosis does not require β-arrestins [59], VPS26 proteins may serve the same role in animals. 

The candidate adaptor VPS26b forms a homodimer or a heterodimer with VPS26a, both required for flg22-mediated internalization of AtRGS1. However, those genes are not involved in AtRGS1 internalization that is induced by high concentrations of glucose [55]. Additionally, the inactivation of three cluster sites (AtRGS1^S428/435/436A^) completely abolishes flg22-induced internalization but only partially affects the glucose-mediated internalization of AtRGS1 [55]. Furthermore, a phosphatase is also required for AtRGS1 stability, and its presence reduces the in vitro identified phosphorylation by the WNKs [60]. These findings suggest an animal-like mechanism in which the phosphorylation patterns are the key for recruitment and posterior signal distinction and transduction.

## 3. Phosphorylation as a Switch Mechanism of AtGPA1

**Figure 3:**
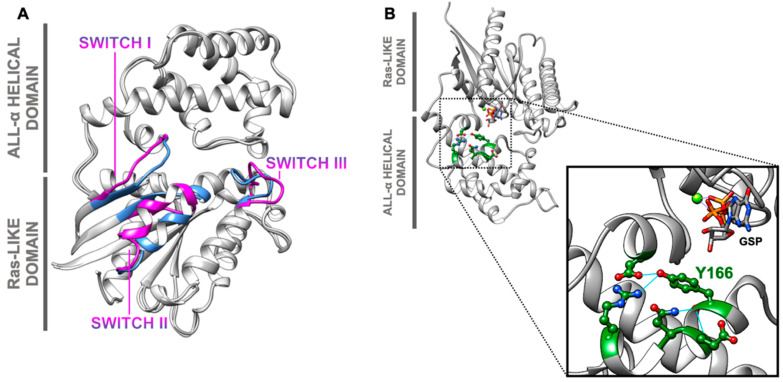
Switching mechanism of Gα. (**A**) The animal Gα activation mechanism. Transducin alpha.GDP (grey and magenta, PDB 1TAG) and transducin alpha.GTP (grey and light blue, PDB 1TND) from *Bos taurus* were selected in order to show nucleotide-induced conformational change in animals. Structures were overlapped, and switch regions of both states were colored in pink and blue, as indicated. Adapted from [67]. (**B**) The plant Gα “phosphoswitch” region. AtGPA1 is phosphorylated at tyrosine 166 in order to affect AtRGS1 interaction and its accelerated GTPase cycle. The crystal structure of AtGPA1 (PDB 2XTZ) is represented in grey with forest green highlights. Tyr166 is at the interface of the two conserved domains and forms hydrogen bonds (cyan) with neighbor residues (balls and sticks). A GTP molecule with Mg^2+^ is near this residue, and they are represented as sticks and as a light green sphere, respectively. Adapted from [68].

Eukaryotic organisms encode over 100 guanine nucleotide-binding proteins (GNBPs), represented by heterotrimeric G proteins, small Ras-related proteins, and translation elongation factors [61]. Besides the high sequence identity, those GNBPs share a common structural core composed of six beta-sheet strands, five alpha-helices, and five highly conserved loop regions that bind to GDP/GTP. Each of the five loops is responsible for phosphate binding, guanine ring binding, or Mg^2+^ binding and coordination [62]. Upon binding, GTP hydrolysis occurs with a subunit-specific intrinsic rate. A conformational change brings the two switch regions (Switch I and Switch II) to a non-flexible conformation that orientates the magnesium ion in order to facilitate the reaction [33,61]. The canonical alpha subunit of heterotrimeric G proteins contains the small Ras-like domain and an all-alpha helical domain that, in animals, is involved in guanine exchange factor (GEF) binding, nucleotide release inhibition, and ubiquitination processes (Figure 3) [62,63,64,65,66].

The *Arabidopsis thaliana* Gα subunit (AtGPA1) has a spontaneous nucleotide exchange activity about 50 times higher than that of Gα_oA_ (G protein alpha subunit o), the fastest exchanging Gα identified in mammalians [13,22]. Even though AtRGS1 maintains AtGPA1 in a resting state by increasing the GTP hydrolysis rate, the endocytosis of the regulator requires prior G-protein activation. Thus, the balance of cycling and hydrolysis within AtGPA1 is crucial for downstream signaling activation [40,68]. There are examples in animals and yeast regarding activation by phosphorylation of Gα. Phosphorylation of the bovine Gsα (Gs alpha subunit) by epidermal growth factor receptor (EGFR) is exclusive to tyrosine residues and promotes adenylate cyclase [69]. In *Saccharomyces cerevisiae*, the alpha subunit Gpa2 is phosphorylated by glycogen synthase kinase (GSK), increasing its localization on the plasma membrane and activating protein kinase A (PKA) at a higher level [70]. Nevertheless, the characterized phosphorylation sites from these events are not conserved among plant components [68]. 

Although only a small amount (4.3%) of phosphopeptides are phosphotyrosines, and there is no evidence of bona fide tyrosine kinases in Arabidopsis [28,71], phosphoproteomics studies have demonstrated a phosphorylation signal at tyrosine 166 of AtGPA1 (Figure 2 and Figure 3B). Furthermore, this residue is one of the BAK1 substrates and has been found to be differentially phosphorylated under abscisic acid (ABA), indole-3-acetic acid (IAA), gibberellic acid (GA), jasmonate (JA), and kinetin treatments [29,68]. The Tyr166 phosphosite is localized in the interface of the two domains, and it is predicted to regulate AtRGS1 binding by forming a salt bridge in this region. AtRGS1 has a higher affinity for the transitional state of alpha, but a phosphomimetic mutation that changes Tyr166 enables AtRGS1 to bind to its GDP-bound state [68]. This new mechanism is dubbed tyrosine phosphoswitching, in which the function of the AtRGS1 protein switches from a GAP (GTPase activating protein) function to a GDI (GDP dissociation inhibitor) function based on the phosphorylation state of its substrate AtGPA1 (Figure 3B). Moreover, flg22 treatment promotes the phosphorylation of AtGPA1 at Thr19, which is essential for RGS1 binding regulation during biotic signaling, and it is also differentially phosphorylated under ABA treatment (Figure 2) [29,33].

The phosphorylation of AtGPA1 under biotic stress and hormone treatment is consistent with the fact that both AtGPA1 and AGB1 interact with the JA signaling regulators TCP14 and JAZ3, transcription factors that are stabilized in the nucleus by both G-subunits ([29,52], internal data). The stabilization of those transcription factors is favored by the phosphorylation of both Tyr166 and the N-terminal residues Ser8, Thr12, Thr15, and Thr19, which promotes the dissociation of AtGPA1 from both AGB and RGS proteins. This mechanism evidences the role of phosphorylated GPA1 during biotic responses and hormone crosstalk, unveiling a novel mechanism of G-protein subunit sequestering for transcriptional regulation [internal data]. Except for Ser8, all involved phosphoresidues were detected in vivo by MS analysis, and Y166 is the most conserved among plants and other eukaryotes (Table 1).

Finally, about 24 residues inside the Ras-like and helical domains have been demonstrated to be phosphorylated in vitro by 11 different RLKs (Table 1). Interestingly, some residues are phosphorylated by different kinases depending on the state of AtGPA1, raising the hypothesis that nucleotide-dependent AtGPA1 conformation is crucial for substrate accessibility and, consequently, for RLK specificity [16].

## 4. Stress Responses through XLG Phosphorylation

The non-canonical Gα subunits called extra-large G proteins (XLGs) are unique to plants [72]. The C-terminal halves of XLG proteins are homologous to those of the canonical alpha subunits. The non-conserved N-terminal halves of XLG proteins contain a nuclear localization signal (NLS) and a cysteine-rich region [73]. This semi-conserved domain lacks many key residues for nucleotide binding, resulting in poor nucleotide affinity and slow GTP hydrolysis [74,75]. In addition, the Arabidopsis XLGs (XLG1, XLG2, and XLG3) can interact with the Gβγ dimer and AtRGS1 under some conditions but with no evidence of an associated GAP activity [72,74].

Multiple data indicate that genetic ablation of *XLGs* results in the opposite effect of ablation of *AtGPA1* regarding pathogen susceptibility, lateral root proliferation, salt stress, and stomatal density [72,73,75,76]. The extra-large subunits are also genetically linked to tunicamycin and D-glucose sensitivity, while *gpa1* mutants display a wild-type phenotype under such treatments [72]. Even though these proteins are thought to be negative regulators of AtGPA1 by sequestering Gβγ or RGS1 from the canonical complex, they may act parallelly during ABA responses and root development [76].

Regarding biotic responses, *xlg2* null mutants have impaired flg22 responses, and both *AtXLG2* and *AtXLG3* genes are induced by this elicitor. In addition, XLG2 and XLG3 interact with BIK1, FLS2, and RbohD (NADPH/respiratory burst oxidase protein D), and the complementation of knockout plants with AtXLG2^S141/148/150/151A^ expression abolishes flg22-induced phosphorylation and lowers ROS response compared to that in wild-type plants [47]. In contrast, XLG2 signaling with CERK1 (CHITIN ELICITOR RECEPTOR KINASE 1) under chitin elicitation is not affected by the same N-terminal mutations [77].

In proteomics studies, XLG2 has several in vivo detected phosphosites: five N-terminal residues respond to ionizing radiation and six respond to “end-of-day” conditions [30,44]. Among these residues, Ser13 responds to osmotic stress, and Ser71/169 respond to nitrate starvation [45,46,48,49,50]. Ser13 and Ser38 display increased phosphorylation signals 15 min after flg22 exposure, while serine residues 75, 185, 190, 191, 194, and 198 show decreased signals after 3 or 15 min of exposure [60]. In addition to the four mutated N-terminal serine residues, XLG2 is differentially phosphorylated at the helical domain (Ser530) by flg22 [51]. Several other phosphorylated sites in the non-conserved region are constitutively detected in different tissues (Table 1). XLG3 has nine N-terminal tissue-specific phosphoresidues under normal conditions [26]. Like XLG2-Ser530 phosphorylation, Ser506 of the XLG3 helical domain is differentially phosphorylated under ABA, sucrose, mannitol, and short cold treatments [42,51,52,53], and it is detected with a reduced signal in the first minutes of flg22 exposure [60].

Although XLG1 has a nuclear localization signal, its localization is partner-dependent [78,79], and it is not phosphorylated at the N-terminus. Instead, it is phosphorylated right after the NLS in the serines 462 and 471 [26,72]. Atypical tyrosine phosphorylation (Y876/879/887) may occur at the end of helix G5 in isoxaben-treated seedlings [42]. Taken together, these data indicate a similar phosphorylation-mediated regulation mechanism between XLG2 and XLG3 under stress responses, but not XLG1, which may be related to its different subcellular localization.

## 5. G**βγ** Specificity and Function

In contrast to being only a negative regulator of Gα signaling, AGB1 is a crucial signaling component in plants [80] like in yeast [81]. Among other phenotypes, *agb1* null mutants exhibit dwarf morphology, impaired abiotic responses, reduced ROS burst under flg22 elicitation, and higher susceptibility to pathogen attack [80,82,83,84,85]. This susceptibility is directly related to the upregulation of JA responsive genes on *agb1* plants, indicating that JA signaling may be negatively regulated by AGB1 [internal data].

Genetic data indicate that AGB1 requires the gamma subunit for signaling. Only AGG1 is linked to pathogen defense, while both AGG1 and AGG2 are involved in auxin-mediated signaling via different mechanisms. The inhibition of germination by D-glucose or osmotic stress is independently mediated by AGG2 or AGG1, respectively [86]. On top of that, AtAGG3 and its rice homologs mediate ion channel regulation, seed, and organ development [87,88]. Consistently with the signaling module, alpha-binding to AGB1 is also gamma-dependent, displaying distinct functions according to its binding partners. While GPA1 has a binding preference for AGB1/AGG3, the interaction of XLG1 and XLG2 with AGB1 depends similarly on AGG1 and AGG2 [72,89]. Additionally, XLG3 binds equally to all three heterodimers and competes with GPA1 for Gβ interaction [72].

The phosphorylation events likely regulate dimer preference and signal specificity since AGB1, AGG2, and AGG3 have MS-confirmed phosphorylation sites [26,42]. The receptor-like kinase complex BAK1/BRI1 interacts with both AGG3 and AGB1, and the latter interaction is increased under 2% D-glucose treatment. Both subunits are phosphorylated by BRI1 in vitro, and inactivation of the corresponding MS-detected sites leads to impaired sugar response in planta [43]. The receptor-like kinase AtZAR1 (ZYGOTIC ARREST 1) has a calmodulin-binding domain, interacts with Gβ, and may integrate Ca^2+^ signaling with the heterotrimeric G-protein pathway [90].

The N-terminal domain of AGB1 has predicted target motifs for glycogen synthase kinase 3/SHAGGY-like protein kinases (GSKs) and interacts with the GSK BIN2. The 3/SHAGGY motifs are present within 46–358 residues, and in vivo phosphorylation of AGB1 has only been detected at Ser2 and Ser4 [26,91]. On top of beta phosphorylation, AGG2 is differentially phosphorylated at non-distinguished serine residues 6, 8, and 9 in response to sucrose and xylanase treatments [25,26,42]. Like in XLG2, an AGG3 phosphosite is identified at Ser37 in response to end-of-day conditions and ionizing radiation (Table 1) [30,44]. Finally, the same site displays an enhanced phosphorylation signal after 15 min of anti-bacterial immunity elicitation [60].

**Figure 4:**
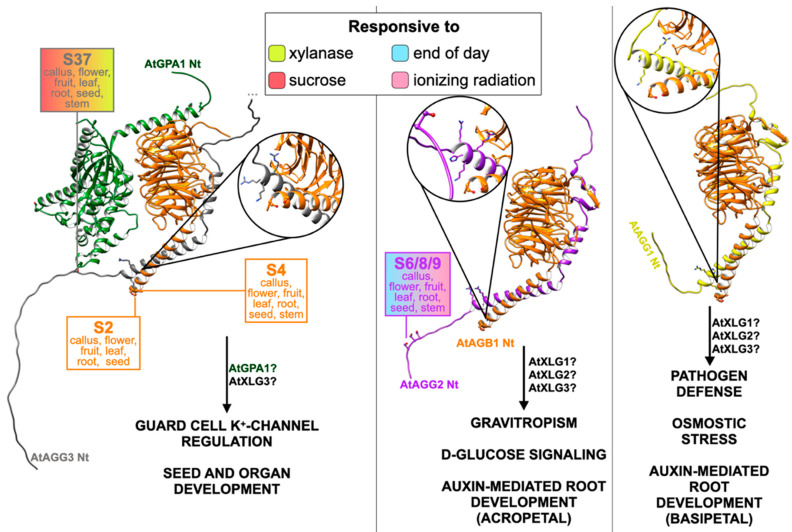
Gβγ specificity and function distinction. AtGPA1 (forest green) binds preferentially to AGB1 (orange) when dimerized with AGG3 (grey), which regulates ion transport, seed, and organ development [72,87,88,89]. β-dimerized AGG2 (purple) binds to the XLGs in order to regulate gravitropism, sugar responses, and root development [72,86]. Phosphorylation occurs in vivo at the N-terminal portions of AGB1, AGG2, and AGG3 [26,42,60]. Beta-gamma complex models were created using AlphaFold2, and top-ranked models were selected for analysis [35]. The heterotrimeric complex was created by overlapping the models with the crystal structure of the heterotrimeric G-protein complex of Bos taurus (PDB 1GOT). Experimental data were obtained from the PhosphAt database (https://phosphat.uni-hohenheim.de, accessed on 23 May 2022) and ATHENA (http://athena.proteomics.wzw.tum.de, accessed on 23 May 2022). AGG3 unmodeled C-terminal regions were removed for better visualization. ATHENA was used to identify tissue-specific phosphorylation, which is pointed out below residue identification. Phosphosites are represented as balls and sticks. Candidate AGB1pS37-interacting residues are represented as sticks only. Color filling indicates experimental treatments.

Molecular protein modeling mapped the beta phosphorylation at or near the Gβγ interaction interface with close (+)-charged residues [35]. Moreover, AGG2 and AGG3 are phosphorylated near these sites and close to the Gα interface. AGG3 shows a long non-structured C-terminal tail (res. 116–251) far from the interface that was excluded from the model for visualization purposes. This structural estimation indicates that phosphorylation may affect the interaction dynamics of the trimer and, therefore, signal specificity (Figure 4).

## 6. G-Paradox and Four-State Model

The nucleotide state of animal Gα modulates the heterotrimer formation from a “switch off” (GDP-bound) to a “switch on” (GTP-bound) structure (Figure 3A) [67]. Thus, it is controversial that, in plants, no structural difference was detected within the trimer during the two nucleotide states of AtGPA1 [92]. Furthermore, genetic complementation of the rice dwarf mutant d1 (OsRGA1-defective) with a constitutive GTP-bound alpha mutant (OsRGA1^Q223L^) rescued the normal development phenotype, suggesting that on–off cycling is not required [93]. Adding XLGs and their functions to the plant G-protein repertoire has moved the plant signaling module even further from the established animal module [72]. Both AtGPA1 and XLGs present nucleotide independency for most functions and structural plasticity [74,89,94].

Another observation is that AtRGS1 strongly controls the complex state in vitro, but *rgs1* plants present subtle phenotypes compared to other G-protein mutants [95]. Furthermore, one of the few *rgs1* strong phenotypes is its poor capability of photosynthetic adjustment under dynamic or excessive irradiation, even though the behavior is wild-type-like during constant light conditions [96]. The RLK phosphorylation over several subunits also differs from the animal paradigm [16,38].

Therefore, to provide a solution to this paradox, the current plant model consists of four described states of Gα—Gα-GTP, Gα-GDP, pGα-GDP, and pGα-GTP—in which only the phosphorylated forms are signaling competent. In addition, RLKs are activated by an external stimulus and phosphorylate RGS1, resulting in an altered GTP/GDP state of the switch. The switch is also phosphorylated by the RLKs, independent of its nucleotide state [95]. Finally, phosphorylation is highlighted as a crucial regulation component, and the post-translational state of the subunits may explain inconsistencies in reverse genetic studies. 

## 7. Conclusions

The phosphorylation at threonine/serine/tyrosine residues modulates many aspects of protein function and, consequently, is a highly regulated process. Advances in protein modeling, genetic data, and phosphoproteomic analysis have provided a direct link between phosphorylation status and G-signaling activation and triggering specificity. Flg22 elicitation induces phosphorylation at Ser428/431 residues on AtRGS1 proteins [27], while glucose induces phosphorylation at Ser428/435/436 [14]. These distinct phosphorylation patterns are implicated in specific cell responses modulated by G-protein activation through different RLKs. AtWNK8 phosphorylates at least two serine residues at the RGS protein upon glucose induction, and this phosphorylation event promotes G-signaling activation and RGS endocytosis [14]. However, FLS2 and its coreceptor BAK1 trigger the phosphorylation of RGS on Ser428/431, promoting its dissociation from FLS2 and Gα [27]. AtGPA1 shows dynamic phosphorylation upon flg22 elicitation, which reduces the phosphorylation level of Thr19, implicating a specific role of this AtGPA1 residue in plant signaling responses to flg22 [33], although the same phosphoresidue is induced by hormone treatment [29]. Therefore, the signaling discrimination relies on a specific combination of phosphorylation between RGS and GPA1 proteins, a regulatory mechanism that may be expanded to form atypical core conformations that include the XLGs and different gamma subunits. 

Herein, we reviewed the phosphorylation status of the G-protein signaling components and its ability to regulate their binding affinity, localization, and stability, thus controlling their function on signal transduction and propagation. However, the characterization of the underlying G-protein phosphorylation status is still in its infancy; hence, the identification of different protein kinase phosphosites might shed light on signal discrimination and G-signaling activation. Furthermore, understanding the underlying mechanism of specific residue phosphorylation can be exploited as a marker for G-protein distinct signaling. Finally, the intricated mechanism of G-protein dynamism in plants does not rely only on a defined composition of the complex or its nucleotide-binding status, but rather is regulated by the phosphorylation status of the main components, RLKs, and other interacting partners, creating a complex post-translational G code for signal transduction.

## Figures and Tables

**Table 1:** MS-detected phosphorylation sites from the Arabidopsis G-protein core.

Protein	Residue	Detected In Vivo?	In Vitro Kinase	Conservation Score (Plants Only) *	Conservation Score (Eukaryotes, Excluding Plants) *
AtRGS1	Ser278	Yes [25]	BRL3, BIK1, PBL1 [15,27]	−0.861	
	Ser339	No [15]	BRL3 [15]	0.714	−1.131
	Ser365	No [15]	BRL3 [15]	−1.373	0.444
	Thr375	No [15]	BRL3 [15]	−1.016	−0.162
	Thr379	No [15]	BRL3 [15]	−0.582	0.483
	Ser405	No [15]	BRL3 [15]	−0.981	0.959
	Ser406	No [15]	BRL3 [15]	−1.139	−0.559
	Ser417	Yes [26]	BRL3, BIK1 [15,27]	1.798	
	Ser428	Yes [26]	BRL3, PEPR1, WNK8, BIK1, PBL1 [11,14,27]	−0.211	
	Ser430	Yes [26,27]	BRL3, BIK1, PBL1 [15,27]	−1.116	
	Ser431	Yes [26,27]	BRL3, BIK1, PBL1 [15,27]	−0.853	
	Ser435	Yes [26]	BRL3, WNK8 [11,14,27]	−1.048	
	Ser436	Yes [26]	BRL3, WNK8 [11,14]	−0.097	
	Ser450	Yes [27]	BIK1, PBL1 [27]	1.297	
	Ser452	Yes [27]	BIK1, PBL1 [27]	1.897	
	Ser453	Yes [27]	BRL3, BIK1, PBL1 [15,27]	0.429	
AtGPA1	Ser8	No [16]	BAK1, PSY1R, PEPR1, BRL3, BRI1, XIP1, AT2G19230, AT2G37050, AT5G62710 [16]	1.567	−0.741
	Thr12	Yes [28,31]	BAK1, SERK1, PSY1R, PEPR1, BRL3, XIP1, AT2G19230, AT2G37050, AT5G62710 [16]	2.432	2.226
	Thr15	Yes [30,32]	BAK1, SERK1, PSY1R, BRI1, XIP1, AT2G19230, AT2G37050, AT5G62710 [16]	3.816	0.489
	Thr19	Yes [29]	BAK1, SERK1, PSY1R, BRL3, BRI1, XIP1, AT2G19230, AT2G37050, AT5G62710 [16]	1.349	0.949
	Ser49	Yes [42]		−0.658	−0.908
	Ser52	No [16]	BRL3, AT2G19230, AT5G62710 [16]	−0.167	−0.945
	Thr53	No [16]	BRI1 [16]	−0.974	−0.942
	Ser73	No [16]	BAK1 [16]	0.293	0.322
	Thr85	No [16]	BAK1, PSY1R, BRL3, BRI1, AT2G19230, AT5G62710 [16]	−0.588	−0.792
	Thr93	No [16]	BAK1, SERK1, PSY1R, BRL3, BRI1, XIP1, AT2G19230 [16]	0.609	−0.700
	Thr101	No [16]	BAK1, XIP1 [16]	5.029	0.514
	Ser103	No [16]	AT2G19230 [16]	−0.179	1.321
	Ser109	No [16]	BAK1, SERK1, BRL3, AT5G62710 [16]	−0.428	1.116
	Ser110	No [16]	BRI1 [16]	5.031	0.509
	Ser112	No [16]	SERK1, AT2G19230, AT2G37050, AT5G62710 [16]	0.333	−0.266
	Thr141	No [16]	BAK1, BRL3 [16]	0.345	1.160
	Thr164	No [16]	SERK1, XIP1, AT5G10290, AT2G37050, AT5G62710 [16]	−0.007	−0.847
	Tyr166	Yes [29]		−0.673	−0.929
	Ser175	No [16]	AT5G62710 [16]	−0.464	0.857
	Thr193	No [16]	BRI1 [16]	−0.985	−0.942
	Thr194	No [16]	BRI1 [16]	−0.680	−0.807
	Ser314	Yes [26]	BAK1, AT5G62710 [16]	0.146	0.303
	Ser315	No [16]	BAK1, AT5G62710 [16]	0.349	−0.304
	Thr339	No [16]	BAK1 [16]	0.079	1.063
	Thr353	No [16]	BRI1 [16]	−0.311	−0.898
AtAGB1	Ser2	Yes [26]		−0.301	1.319
	Ser4	Yes [26]		2.106	1.568
	Thr14	No [43]	BRI1 [43]	1.347	−0.356
	Thr16	No [43]	BRI1 [43]	0.838	−0.137
	Thr34	No [43]	BRI1 [43]	−0.110	−0.003
	Ser40	No [43]	BRI1 [43]	0.520	0.002
	Thr46	No [43]	BRI1 [43]	2.140	0.422
	Ser49	No [43]	BRI1 [43]	1.972	0.304
	Thr53	No [43]	BRI1 [43]	0.048	1.096
	Thr65	No [43]	BRI1 [43]	0.034	−0.538
	Ser70	No [43]	BRI1 [43]	−0.421	−0.529
	Ser82	No [43]	BRI1 [43]	−1.179	−0.624
	Thr100	No [43]	BRI1 [43]	0.228	−0.127
	Thr243	No [43]	BRI1 [43]	−0.687	−0.561
	Thr253	No [43]	BRI1 [43]	0.776	−0.327
AtAGG2	Ser6	Yes [26]		1.889	−0.927
	Ser8	Yes [25]		0.223	−0.428
	Ser9	Yes [42]		1.827	0.045
AtAGG3	Ser21	No [43]	BRI1 [43]	−0.967	1.287
	Ser22	No [43]	BRI1 [43]	−0.933	0.097
	Ser37	Yes [26]	BRI1 [43]	−1.522	1.643
	Ser78	No [43]	BRI1 [43]	1.621	−2.114
	Thr92	No [43]	BRI1 [43]	0.913	−1.267
AtXLG1	Ser462	Yes [26]		1.114	3.103
	Ser471	Yes [26]		0.233	1.061
	Tyr876	Yes [42]		1.458	2.004
	Tyr879	Yes [42]		0.231	1.367
	Tyr887	Yes [42]		−0.188	−0.128
AtXLG2	Ser13	Yes [30,41,44]		0.644	
	Ser23	Yes [30,45,46]		1.892	
	Ser38	Yes [26]		−0.937	
	Ser69	Yes [47]		0.404	
	Ser71	Yes [48]		0.556	
	Ser72	Yes [47]		0.542	
	Ser75	Yes [30,44]		0.689	
	Ser141	Yes [26]		1.825	
	Ser148	Yes [47]	BIK1 [47]	−0.079	
	Ser150	Yes [47]	BIK1 [47]	1.152	
	Ser151	Yes [30,44]		1.467	
	Ser154	Yes [30,44]		1.143	
	Ser156	Yes [47]		1.919	
	Ser169	Yes [30,44,46,48,49,50]		0.681	
	Ser191	Yes [47]		0.865	
	Ser194	Yes [26]		1.539	
	Ser489	Yes [47]		−0.520	−1.243
	Ser530	Yes [51]		0.991	0.644
	Thr773	Yes [47]		0.655	0.550
	Ser774	Yes [47]		0.190	−0.397
AtXLG3	Ser78	Yes [26]		1.823	
	Ser82	Yes [26]		−0.216	
	Ser85	Yes [26]		0.112	
	Ser99	Yes [26]		1.173	
	Ser101	Yes [26]		1.432	
	Ser103	Yes [26]		−0.082	
	Ser107	Yes [26]		−0.421	
	Ser243	Yes [26]		−0.533	
	Ser416	Yes [26]		0.247	−1.125
	Ser506	Yes [52,53]		0.846	−1.221

* Normalized conservation score obtained from the ConSurf server. A lower score indicates higher residue conservation. Sequences were obtained using the BLAST tool (https://blast.ncbi.nlm.nih.gov/Blast.cgi?PAGE=Proteins, accessed on 20 May 2022), and representative sequences were selected using CD-HIT (http://weizhong-lab.ucsd.edu/cdhit_suite/, accessed on 20 May 2022) with a sequence identity cut-off of 0.9. MSA was obtained with ClustalOmega (https://www.ebi.ac.uk/Tools/msa/clustalo/, accessed on 20 May 2022). For non-plant eukaryotic conservation, RGS (PF00615) and Gγ (PF00631) family sequences were obtained from Pfam. AtRGS1 and XLGs’ non-conserved regions were excluded from the final analysis.

## Data Availability

The in vivo data analyzed in this study is available online at the Arabidopsis Protein Phosphorylation Site Database and at the Arabidopsis THaliana ExpressioN Atlas.

## Glossary

GPA1	Heterotrimeric G-protein Alpha Subunit
AGB1	Heterotrimeric G-protein Beta Subunit
AGG	Heterotrimeric G-protein Gamma Subunit
RGS	Regulator of G Signaling
XLG	Extra-Large G Protein
CME	Clathrin-Mediated Endocytosis
SDE	Sterol-Dependent Endocytosis
GPCR	G-Protein-Coupled Receptor
GEF	Guanine Nucleotide Exchange Factor
RLK	Receptor-Like Kinase
V_2_R	V2 Vasopressin Receptor
GRK	GPCR Kinase
FLS2	FLAGELLIN-SENSITIVE 2
BAK1	BRI1-ASSOCIATED RECEPTOR KINASE 1
NFR1	Nod Factor Receptor 1
ABA	Abscisic Acid
VPS26	Vacuolar Sorting Proteins 26
WNK	WITH NO LYSINE KINASE
GNBP	Guanine Nucleotide-Binding Protein
EGFR	Epidermal Growth Factor Receptor
GSK	Glycogen Synthase Kinase
PKA	Protein Kinase A
IAA	Indole-3-Acetic Acid
CERK1	Chitin Elicitor Receptor Kinase 1
JA	Jasmonic Acid
GA	Gibberellic Acid
GAP	GTPase Activating Protein
MS	Mass Spectrometry
